# Dietary Intake Assessment Using a Novel, Generic Meal–Based Recall and a 24-Hour Recall: Comparison Study

**DOI:** 10.2196/48817

**Published:** 2024-02-14

**Authors:** Cathal O'Hara, Eileen R Gibney

**Affiliations:** 1 University College Dublin Institute of Food and Health Science Centre South University College Dublin Dublin Ireland; 2 Insight Centre for Data Analytics University College Dublin Belfield Dublin Ireland; 3 School of Agriculture and Food Science University College Dublin Belfield Dublin Ireland

**Keywords:** meal patterns, eating behaviors, eating occasions, nutrition assessment, dietary intake assessment, 24-hour recall, relative validity

## Abstract

**Background:**

Dietary intake assessment is an integral part of addressing suboptimal dietary intakes. Existing food-based methods are time-consuming and burdensome for users to report the individual foods consumed at each meal. However, ease of use is the most important feature for individuals choosing a nutrition or diet app. Intakes of whole meals can be reported in a manner that is less burdensome than reporting individual foods. No study has developed a method of dietary intake assessment where individuals report their dietary intakes as whole meals rather than individual foods.

**Objective:**

This study aims to develop a novel, meal-based method of dietary intake assessment and test its ability to estimate nutrient intakes compared with that of a web-based, 24-hour recall (24HR).

**Methods:**

Participants completed a web-based, generic meal–based recall. This involved, for each meal type (breakfast, light meal, main meal, snack, and beverage), choosing from a selection of meal images those that most represented their intakes during the previous day. Meal images were based on generic meals from a previous study that were representative of the actual meal intakes in Ireland. Participants also completed a web-based 24HR. Both methods were completed on the same day, 3 hours apart. In a crossover design, participants were randomized in terms of which method they completed first. Then, 2 weeks after the first dietary assessments, participants repeated the process in the reverse order. Estimates of mean daily nutrient intakes and the categorization of individuals according to nutrient-based guidelines (eg, low, adequate, and high) were compared between the 2 methods. *P* values of less than .05 were considered statistically significant.

**Results:**

In total, 161 participants completed the study. For the 23 nutrient variables compared, the median percentage difference between the 2 methods was 7.6% (IQR 2.6%-13.2%), with *P* values ranging from <.001 to .97, and out of 23 variables, effect sizes for the differences were small for 19 (83%) variables, moderate for 2 (9%) variables, and large for 2 (9%) variables. Correlation coefficients were statistically significant (*P*<.05) for 18 (78%) of the 23 variables. Statistically significant correlations ranged from 0.16 to 0.45, with median correlation of 0.32 (IQR 0.25-0.40). When participants were classified according to nutrient-based guidelines, the proportion of individuals who were classified into the same category ranged from 52.8% (85/161) to 84.5% (136/161).

**Conclusions:**

A generic meal–based method of dietary intake assessment provides estimates of nutrient intake comparable with those provided by a web-based 24HR but with varying levels of agreement among nutrients. Further studies are required to refine and improve the generic recall across a range of nutrients. Future studies will consider user experience including the potential feasibility of incorporating image recognition of whole meals into the generic recall.

## Introduction

Well-established causal relationships exist between dietary intakes and health [1]. Accurate dietary intake assessment is required to identify suboptimal intakes and devise interventions to address them [2]. Existing food-based methods of dietary intake assessment can be time-consuming and burdensome for individuals to complete [3]. In addition, not all methods provide information such as the timing of meals, different foods that are consumed in combination as part of these meals, or combinations of different meals over a day; for example, the food frequency questionnaire (FFQ) focuses on mean daily food and nutrient intakes [4].

The time and effort required for individuals to complete the existing methods of dietary intake assessment such as FFQs, 24-hour recalls (24HRs), and food diaries may limit adherence to and engagement with such methods, which are often used on web-based and mobile-based personalized nutrition platforms [3]. A survey of 2382 adults across Europe found that ease of use was the most important feature for participants when choosing a nutrition or diet app [5]. Digital versions of 24HRs and food diaries require the user to text search for a food and then select from a list of results the food that they consumed. This process is then repeated for each food in the meal and each meal in the day [6]. Intakes of whole meals can be recorded in a manner that is less burdensome than recording individual foods, providing a potentially low-burden method for dietary intake assessment [3]. For example, instead of text searching for individual food, as is required in 24HRs and food diaries, the user could be presented with images of whole meals and choose the image most similar to their meal [3]. The use of meal-based methods may also be preferred in personalized nutrition because people tend to perceive their dietary intakes in terms of the meals they have consumed rather than their daily intakes of nutrients or foods; therefore, recording dietary intakes and providing dietary advice in this manner may be more intuitive [7,8].

Although the number of studies examining meal patterns has increased in recent years [4], only 3 studies [9-11] have developed meal-based methods of dietary intake assessment rather than using existing food-based methods. Englund-Ögge et al [9] used a method in which participants reported how often they consumed various meal types (breakfast, morning snack, lunch, afternoon snack, dinner, evening snack, supper, and night meal). Wilson et al [10] used a similar approach, but instead of using meal types, they divided the day into periods and asked participants to report for each period whether they consumed nothing, a snack, a small meal, or a large meal and whether they drank nothing, alcohol, water, or something else. These approaches to meal-based dietary assessment are simple to complete and provide qualitative information about meal types and their timing. They do not, however, provide the qualitative detail necessary to identify the different combinations of foods being consumed in meals and the combinations of those meals over a day or the quantitative detail required to estimate the nutrient intakes arising from those consumptions. Murakami et al [11] developed an approach that involves participants reporting the frequency of consumption of combinations of food groups and foods at specified meal types (breakfast, morning snack, lunch, afternoon snack, dinner, and night snack), and it has been designed for use in the Japanese population. This approach allows for the identification of meal patterns and nutrient intakes but still requires individuals to report intakes at the food level. None of those studies, however, allow for the reporting of meal portion sizes or capture information from the previous 24 hours in the form of recall.

Several tools have been developed that use image recognition software for dietary intake assessment [12]. However, these methods remain food based rather than meal based. The software first segments a meal image into its constituent foods and then provides a suggested match for each food in the image. The user must then confirm whether the suggested foods are correct. For any missing or incorrect foods, the user must text search for the correct food and add it to their record [12,13]. No study that allows individuals to record their dietary intakes at the meal level by reporting intakes of whole meals rather than individual foods or food groups has been identified.

This study aimed to develop a novel, meal-based method of dietary intake assessment that would allow individuals to report their intakes of whole meals rather than reporting the individual foods that make up those meals and to compare this method with a web-based 24HR.

## Methods

### Ethical Considerations

The human research ethics committee of University College Dublin granted ethics approval to conduct this study (LS-21-64-OHara-Gibney). Participants were assigned a study number, and on completion of data collection any information linking this number to participants’ personal data was deleted, thus de-identifying participants. No financial compensation was provided for participation in this study. However, on completion of the study, all participants received a personalized nutrition advice report based on the data they provided during the 24HRs.

### Recruitment

The target sample size was 160 participants, based on a previous review of comparisons between digital and paper-based 24HRs, which found a range of sample sizes from 53 to 167 [14]. There were no studies of meal-based methods of dietary assessment on which to base the sample size.

**Figure 1:**
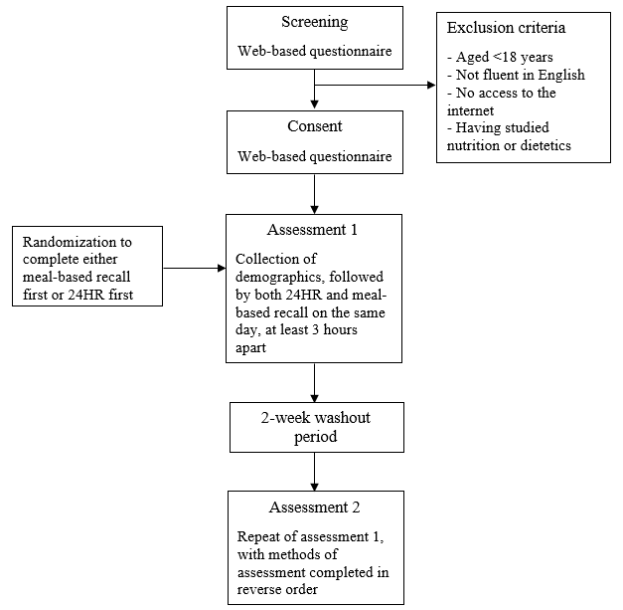
Flow diagram of participants’ journey through the study. 24HR: 24-hour recall.

Recruitment was conducted using local radio, local newspaper, posters, social media, and word of mouth. Researchers directed potential participants to a web page containing full details of the study requirements and contact details of the researchers for further queries, if required. After reading the study information, potential participants could indicate whether they had read and understood the material and agree or disagree to proceed to the web-based screening questionnaire to determine their eligibility to participate in the study. Those who were eligible then completed a web-based consent form to provide electronic informed consent. People were eligible if they were aged >18 years, were fluent in English, had regular access to the internet, and were not current or former students of a degree in nutrition or dietetics (Figure 1).

### Study Design

Participants who were deemed eligible to participate and provided consent were contacted via email, which provided details about the next steps of the study and the links to the 2 web-based dietary intake assessment tools (24HR and generic meal–based recall). A crossover design was used with regard to the order in which participants completed the recalls. Participants were randomized to complete either one of the 2 methods first and then complete the second method at least 3 hours later on the same day. Participants were also randomized regarding whether they would recall a weekday or a weekend day. Then, 2 weeks after having completed the first set of recalls, participants completed the recalls again in the reverse order (compared with the order in which they were completed on the first occasion), followed by the completion of the evaluation questionnaire (Figure 1).

### Overview of the Generic Meal–Based Dietary Recall

**Figure 2:**
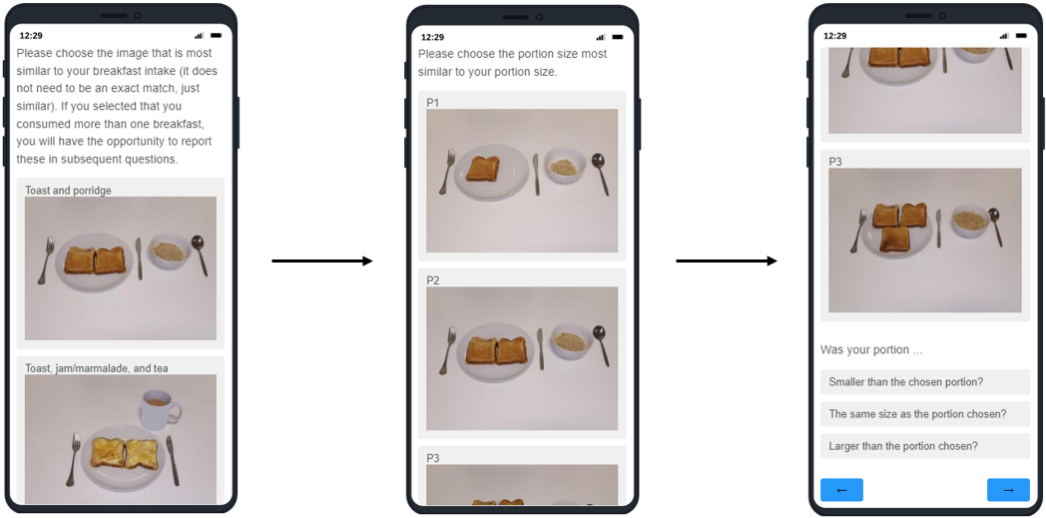
An example of the user interface of the generic meal–based recall, where participants are asked to choose the generic breakfast image that most represents their breakfast intake on the previous day, to specify the portion size that they had consumed for their chosen breakfast meal, and to answer the follow-up portion size question.

The meal-based dietary intake assessment method was administered using Qualtrics XM (Qualtrics International Inc), a web-based platform for questionnaires. Before completing the dietary intake assessment, participants provided information about their sex, age, weight, and height via a web-based questionnaire. Participants were then asked to select the meal types they had consumed on the previous day from the following list: breakfast, morning snack, lunch, afternoon snack, evening meal, evening snack, and beverage (for beverages consumed alone without food). For each meal type selected, participants were presented with a series of images of generic meals that are associated with that meal type and asked to choose the meal image that was most similar to the meal that they had consumed (Figure 2). For each selected meal, participants were asked to choose from 3 different images of that meal, each representing a different portion size, and then asked to select whether the chosen image was smaller than the chosen portion, the same size as the portion chosen, or larger than the portion chosen (Figure 2). For beverages, participants were asked to choose from 3 different images of that beverage, representing 3 different portion sizes, and then asked to select the number of those portion sizes that they had consumed. For each meal type, participants could choose the option that none of the images presented were representative of their intake for that meal type, for example, “none of the above options are similar to what I ate for breakfast.” If participants selected this option, a box appeared, in which they could enter a free-text description of what they had consumed for that meal. This allowed the researchers to determine whether a suitable generic meal could have been chosen or whether there was, in fact, no matching generic meal.

### Development of the Meal-Based Dietary Recall

The process of identifying the generic meals that were presented to participants as images has been described in detail elsewhere [15]. In brief, data from the Irish National Adult Nutrition Survey (NANS; 2008-2010) [16] were used. This is a representative data set about dietary intakes of 1500 adults in Ireland, collected using 4-day weighed food diaries. The meals reported were categorized into the following meal types: breakfast, light meals, main meals, snacks, and beverages. A nutrient profiling score, the Nutrient Rich Foods (NRF) Index [17], was calculated for each meal; specifically, the NRF9.3 version of that profiling score was used. Within each meal type, meals were grouped (ie, clustered) using partitioning around the medoids clustering to identify groups of meals that had similar NRF Index scores and food group composition. These groups were defined as generic meals.

The 27,336 individual meals consumed by the participants in the NANS study were condensed to 63 generic meals; 49% (31/63) of these were consumed during the week and 51% (32/63) during the weekend. Among the 63 generic meals that were identified, there was overlap between weekday and weekend meals. That is, some weekday meals were the same as the weekend meals. When these duplicates were removed, participants were presented with 43 meal images: 5 (12%) breakfasts, 5 (12%) snacks (repeated for morning, afternoon, and evening snacks), 10 (23%) lunches, 19 (44%) dinners, and 4 (9%) beverages.

The nutrient content of a given generic meal was defined as the mean nutrient content of the individual meals that made up that generic meal per 100 g. Each generic meal was assigned 7 portion sizes by ordering each of the individual meals by weight and dividing the meals into 7 equal parts based on septile values for meal weight. The median weights for each part were assigned as the generic portion size for that meal. The nutrient composition for each of the portion sizes was calculated using the meal weight for that portion and the generic meal nutrient composition [15]. Within the meal intake assessment tool presented in this study, the second, fourth, and sixth portion sizes were used as the 3 portion size images shown to participants, with the options asking whether the image chosen was smaller than, the same as, or larger than that consumed, allowing participants to be categorized as consuming the first, third, fifth, or seventh portion size for a given meal.

### 24HR Method

Participants completed their 24HRs using a validated, web-based, self-administered 24HR tool called Foodbook24, which follows the multipass recall method [18-20]. Participants first chose the meal types that they had consumed from the following list: breakfast, morning snack, lunch, afternoon snack, evening meal, and evening snack with the option to add additional snacks. For each of the selected meal types, participants added the foods and beverages they had consumed as part of those meal types by text searching from the food list using a search bar. Portion size was then reported based on the number of the food or beverage items consumed or from portion size photographs, as appropriate. Participants were then presented with the list of foods they had recorded for review, before being presented with a list of commonly forgotten foods. The food list contained food composition data from the McCance and Widdowson’s Composition of Foods Integrated Dataset (CoFID) [21], with some additions relevant to dietary intakes in Ireland. The development of Foodbook24 and its food list is described in detail elsewhere [18,20].

### Statistical Analysis

All analyses were performed using R (version 4.2.2, R Foundation for Statistical Computing) [22] in the RStudio integrated development environment (version 2022.07.2+576, Posit PBC) [23]. Data from 24HRs were used to identify participants likely to be misreporters of energy intake (EI), based on the ratio of estimated EI to basal metabolic rate (BMR; EI:BMR) using the BMR equations from Henry [24]. On the basis of the Goldberg equations [25], EI:BMR <0.96 was deemed indicative of underreporting, and EI:BMR >2.49 was indicative of overreporting. The analysis presented in this paper includes all participants (161/161, 100%), given the negligible differences observed when misreporters were removed; the analysis of the smaller data set with misreporters excluded is provided in Multimedia Appendix 1. *P* values of <.05 were considered statistically significant.

The Shapiro-Wilk test was used to determine whether the differences in the variables between methods were normally distributed and confirmed using visual inspection of histograms. Wilcoxon signed rank test was performed to compare nutrient intake estimates obtained from the web-based 24HR with those obtained from the generic meal–based recall. Wilcoxon effect size (*r*) was calculated. Effect size ≥0.1 and <0.3 was considered small, effect size ≥0.3 and <0.5 was considered moderate, and effect size ≥0.5 was considered large [26]. Bland-Altman analysis was also performed, whereby the mean difference between the 2 data sets and the limits of agreement (LOAs; mean difference – 1.96 SD to mean difference + 1.96 SD) for each nutrient were calculated. The correlation of nutrient intakes between the 2 methods was assessed using Spearman rank correlation coefficients. Correlation coefficient <0.20 was indicative of poor correlation, coefficient ≥0.20 and <0.50 was indicative of acceptable correlation, and coefficient ≥0.5 was indicative of good correlation [27].

Cross-classification of quartiles was performed for all nutrients (23/23, 100%) assessed. That is, nutrient intakes from both methods were divided into quartiles to determine the proportion of participants who remained in the same quartile for both methods (exact agreement), the proportion of participants who were classified in the same or adjacent quartiles (exact + adjacent), the proportion of participants who were classified 2 quartiles apart (disagreement), and the proportion of participants who were classified 3 quartiles apart (extreme disagreement). Participants were also classified according to nutrient-based dietary guidelines, separately for both methods [28-30]. For example, they were classified based on whether their nutrient intakes were low, adequate, or high according to those guidelines. The nutrients assessed were those deemed to be of public health relevance and included protein, carbohydrate, fat, monounsaturated fat, polyunsaturated fat, saturated fat, salt, dietary fiber, calcium, iron, folate, thiamin, riboflavin, and vitamin C. The proportion of individuals who were classified into the same category based on both methods was calculated for each nutrient.

### Evaluation Questionnaire

Participants also completed an evaluation questionnaire at the end of the study, which was administered via Qualtrics XM (Qualtrics International Inc). Participants were asked to what extent they agreed that the meals presented in the meal-based dietary intake assessment tool were representative of what they consume, that the portion sizes presented were representative of what they consume, that the instructions were clear and easy to understand, and that overall, the tool was easy to use. The response options were agree, somewhat agree, somewhat disagree, or disagree. Participants were also asked how they would describe the ease of use of the meal-based tool compared with the 24HR with the response options being: better, somewhat better, somewhat worse, or worse. Finally, participants were asked to respond either yes or no as to whether they would consider using a similar tool to the meal-based tool in the future.

## Results

### Study Sample

A total of 161 participants completed both methods of dietary intake assessment at 2 time points. Most participants were female (131/161, 81.4%), the median age was 54 (IQR 39-63) years, and the median BMI was 25.3 (IQR 22.5-28.9) kg/m^2^ (Table 1).

**Table 1:** Participant demographics and anthropometry^a^.

Characteristics	Female participants (n=131, 81.4%), median (IQR)	Male participants (n=30, 18.6%), median (IQR)	Total (N=161), median (IQR)
Age (y)	54 (41-63)	54 (37-63)	54 (39-63)
Weight (kg)	67.9 (60.5-75.6)	80.8 (75.6-94.0)	70 (62-80.8)
Height (m)	1.63 (1.59-1.68)	1.8 (1.75-1.83)	1.65 (1.60-1.74)
BMI (kg/m^2^)	25.2 (22.5-28.8)	26.0 (22.9-28.8)	25.3 (22.5-28.9)

^a^Weight and height were self-reported by participants, and BMI was subsequently calculated from the self-reported values.

### Daily Nutrient Intakes

For the 23 variables compared, the percentage difference between the meal-based and 24HR methods ranged from 0% to 46.7%, with the median percentage difference being 7.6% and with the generic method providing a higher estimate than the 24HR for 18 nutrients. *P* values for the differences between the 2 methods ranged from <.001 to .97, with 13 (57%) of 23 comparisons reaching statistical significance (*P*<.05). Among the 23 variables, effect sizes for the differences were small for 19 (83%) variables, moderate for 2 (9%) variables (folate in µg and sodium in mg), and large for 2 (9%) variables (polyunsaturated fat in g and as % total EI [TEI]; Table 2).

**Table 2:** Median (IQR) daily nutrient intakes estimated using the web-based, 24-hour recall (24HR) and novel, generic meal–based recall^a,b^.

Nutrient intake	Recall method, median (IQR)		Difference (%)	*P* value	Effect size (*r*)	Effect size (magnitude)
	24HR	Generic				
Energy (kcal)	1569.9 (1318.9-2005.7)	1715.3 (1440.9-1935.3)	9.3	.18	0.107	Small
Protein (g)	65.4 (51.4-86.8)	72.7 (63.2-82.1)	11.2	.69	0.032	Small
Protein (% TEI^c^)	16.6 (13.8-20.1)	16.8 (15.8-18.3)	0.8	.45	0.060	Small
Carbohydrate (g)	192 (140.5-243.3)	202.8 (170.3-240)	5.6	.04	0.162	Small
Carbohydrate (% TEI)	44.5 (39.4-50.4)	45.5 (43.7-47.4)	2.3	.15	0.113	Small
Sugar (g)	71.8 (49.5-97.7)	84.8 (64.3-102.4)	18.1	.001	0.263	Small
Sugar (% TEI)	16.6 (12.8-21.4)	18.6 (16.8-21)	12.2	.002	0.249	Small
Dietary fiber (g)	17.2 (12.3-21.7)	16.7 (14.6-19.8)	−3.4	.24	0.093	Small
Total fat (g)	61.6 (47.1-77.7)	63.1 (52.8-73.5)	2.4	.89	0.011	Small
Total fat (% TEI)	34.7 (29.4-40.2)	33.8 (31.8-35.4)	−2.5	.03	0.169	Small
Saturated fat (g)	22 (15.7-30)	25.1 (21.3-29.3)	14.2	.01	0.202	Small
Saturated fat (% TEI)	12.3 (9.3-15)	13.5 (12.9-14.2)	9.8	.005	0.220	Small
Monounsaturated fat (g)	22.1 (15.4-29.3)	22.7 (19-26.3)	2.7	.97	0.004	Small
Monounsaturated fat (% TEI)	12.3 (10.4-14.7)	12.1 (11.2-12.8)	−1.5	.33	0.076	Small
Polyunsaturated fat (g)	7.6 (5.7-9.7)	11.1 (9.2-12.8)	46.7	<.001	0.598	Large
Polyunsaturated fat (% TEI)	4.2 (3.4-5)	5.9 (5.5-6.2)	41	<.001	0.723	Large
Vitamin D (µg)	2.2 (0.8-4.2)	2.3 (1.9-2.6)	7.6	.15	0.113	Small
Folate (µg)	215.1 (173.4-279.8)	198.1 (165.5-223.4)	−7.9	<.001	0.334	Moderate
Vitamin C (mg)	72.3 (32.4-123.3)	67.1 (55.1-80.2)	−7.2	.002	0.244	Small
Calcium (mg)	686 (509.4-884.2)	796.4 (670.3-944)	16.1	.002	0.250	Small
Iron (mg)	9.9 (7.7-12.6)	9.9 (8.1-12)	0	.13	0.119	Small
Potassium (mg)	2865.5 (2095.8-3462.8)	2716.2 (2200.3-3066.4)	−5.2	.01	0.204	Small
Sodium (mg)	1685.5 (1157-2142.6)	2161 (1853.1-2424.3)	28.2	<.001	0.398	Moderate

^a^*P* values were derived using Wilcoxon signed rank test, with *P*<.05 indicating statistical significance.

^b^Effect size ≥0.1 and <0.3 was considered small, effect size ≥0.3 and <0.5 was considered moderate, and effect size ≥0.5 was considered large [26].

^c^TEI: total energy intake.

**Figure 3:**
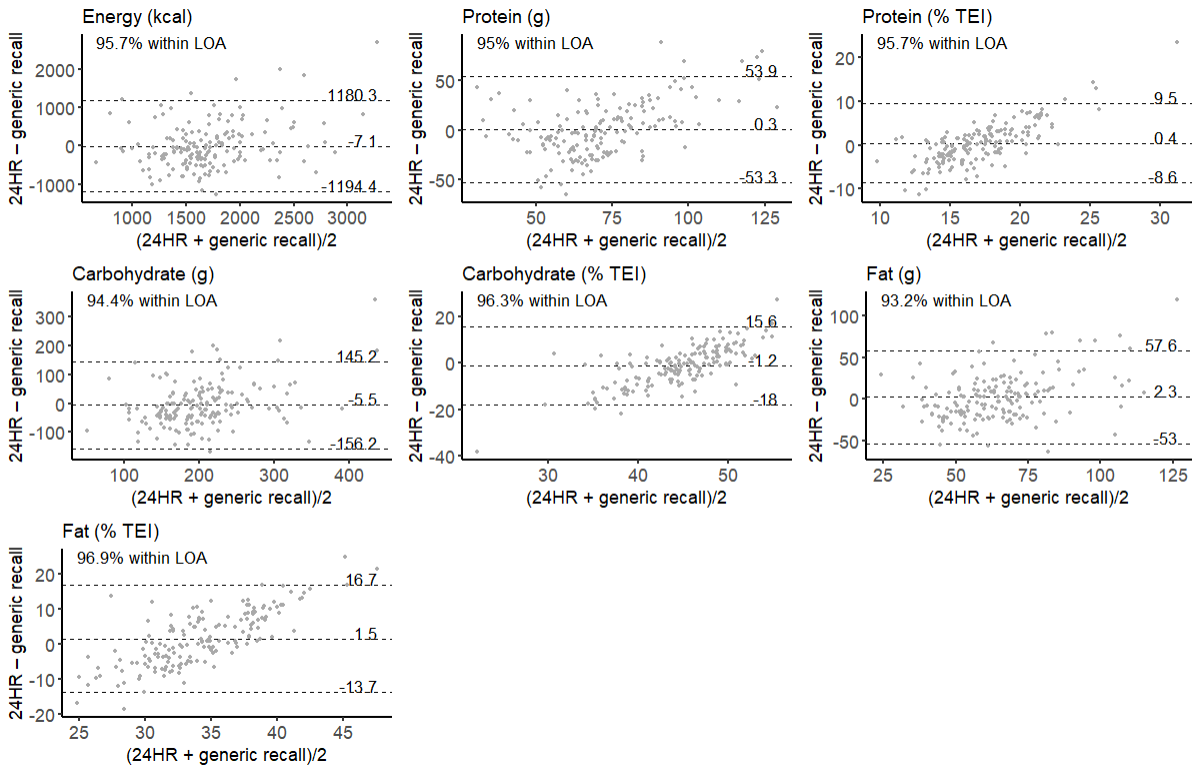
Bland-Altman plots of energy and macronutrient intake estimates. The middle, dashed line and associated number represents the mean difference in daily intakes between web-based 24-hour recall (24HR) and generic recall. The upper and lower dashed lines and associated numbers represent the upper and lower limits of agreement (LOAs), respectively. Each point represents an individual participant (N=161). TEI: total energy intake.

Comparing the differences using the Bland-Altman analysis, the mean differences between the 2 methods for macronutrients were close to 0, whereas those for micronutrients were larger. The LOAs tended to be wide for all nutrients (23/23, 100%). The analysis identified 17 (74%) nutrients for which ≥95% of participants fell within the LOA. The proportion of individuals who fell within the LOA ranged from 92.5% (149/161) for polyunsaturated fats (% TEI) to 98.1% (158/161) for dietary fiber (g). Bland-Altman plots for energy and macronutrients are presented in Figure 3; values from the Bland-Altman analysis for the remaining nutrients are given in Table 3.

**Table 3:** Bland-Altman analysis of nutrient intake estimates obtained from the web-based, 24-hour recall and the generic recall^a^.

Nutrient intake	Mean difference (SD)	Lower LOA^b^	Upper LOA	Participants within LOA (N=161), n (%)
Sugar (g)	−6.2	−89.6	77.1	152 (94.4)
Sugar (% TEI^c^)	−1.3	−14.6	12	155 (96.3)
Dietary fiber (g)	1.4	−16	18.8	158 (98.1)
Saturated fat (g)	−1.7	−26	22.6	152 (94.4)
Saturated fat (% TEI)	−0.8	−9.3	7.7	155 (96.3)
Monounsaturated fat (g)	0.8	−22.3	24	157 (97.5)
Monounsaturated fat (% TEI)	0.4	−6.4	7.2	155 (96.3)
Polyunsaturated fat (g)	−3.0	−11.9	6	151 (93.8)
Polyunsaturated fat (% TEI)	−1.5	−4.9	1.8	149 (92.5)
Vitamin D (µg)	0.8	−5.3	6.8	154 (95.7)
Folate (µg)	43.9	−176.6	264.4	152 (94.4)
Vitamin C (mg)	26.3	−124.3	177	154 (95.7)
Calcium (mg)	−54.2	−765	656.6	155 (96.3)
Iron (mg)	0.6	−7.9	9.2	152 (94.4)
Potassium (mg)	252.2	−1733.8	2238.2	150 (93.2)
Sodium (mg)	−347.6	−2266.3	1571.2	153 (95)

^a^Differences are given as values from the 24-hour recall minus values from the generic recall.

^b^LOA: limit of agreement.

^c^TEI: total energy intake.

Correlation coefficients were statistically significant (*P*<.05) for 18 (78%) of the 23 variables. No significant correlation was identified for total fat (% TEI), monounsaturated fat (% TEI), polyunsaturated fat (% TEI), vitamin D (µg), and sodium (mg). For those where a statistically significant correlation was identified, they ranged from 0.16 for saturated fat as % TEI to 0.45 for sugar in grams, with median correlation of 0.32 (IQR 0.25-0.40; Table 4).

**Table 4:** Comparison of daily nutrient intakes estimated using the web-based, 24-hour recall and the novel, meal-based method, based on correlation and cross-classification of quartiles.

Nutrient intake	Correlation			Cross-classification of quartiles (N=161)			
	Spearman coefficient	*P* value	Exact agreement, n (%)		Exact + adjacent, n (%)	Disagreement, n (%)	Extreme disagreement, n (%)
Energy (kcal)	0.34	<.001	57 (35.4)		115 (71.4)	34 (21.1)	12 (7.5)
Protein (g)	0.26	.001	56 (34.8)		112 (69.6)	41 (25.5)	8 (5)
Protein (% TEI^a^)	0.43	<.001	59 (36.6)		129 (80.1)	26 (16.1)	6 (3.7)
Carbohydrate (g)	0.42	<.001	62 (38.5)		127 (78.9)	25 (15.5)	9 (5.6)
Carbohydrate (% TEI)	0.33	<.001	51 (31.7)		122 (75.8)	28 (17.4)	11 (6.8)
Sugar (g)	0.45	<.001	53 (32.9)		126 (78.3)	26 (16.1)	9 (5.6)
Sugar (% TEI)	0.40	<.001	55 (34.2)		123 (76.4)	32 (19.9)	6 (3.7)
Dietary fiber (g)	0.43	<.001	57 (35.4)		125 (77.6)	26 (16.1)	10 (6.2)
Total fat (g)	0.24	.002	53 (32.9)		113 (70.2)	36 (22.4)	12 (7.5)
Total fat (% TEI)	0.14	.07	37 (23)		102 (63.4)	46 (28.6)	13 (8.1)
Saturated fat (g)	0.20	.01	53 (32.9)		112 (69.6)	38 (23.6)	11 (6.8)
Saturated fat (% TEI)	0.16	.045	41 (25.5)		110 (68.3)	38 (23.6)	13 (8.1)
Monounsaturated fat (g)	0.20	.01	53 (32.9)		112 (69.6)	36 (22.4)	13 (8.1)
Monounsaturated fat (% TEI)	0.02	.75	42 (26.1)		97 (60.2)	41 (25.5)	23 (14.3)
Polyunsaturated fat (g)	0.18	.02	52 (32.3)		108 (67.1)	41 (25.5)	12 (7.5)
Polyunsaturated fat (% TEI)	0.13	.11	47 (29.2)		112 (69.6)	32 (19.9)	17 (10.6)
Vitamin D (µg)	0.14	.07	50 (31.1)		104 (64.6)	35 (21.7)	22 (13.7)
Folate (µg)	0.31	<.001	51 (31.7)		114 (70.8)	34 (21.1)	13 (8.1)
Vitamin C (mg)	0.34	<.001	42 (26.1)		111 (68.9)	35 (21.7)	15 (9.3)
Calcium (mg)	0.26	.001	57 (35.4)		114 (70.8)	34 (21.1)	13 (8.1)
Iron (mg)	0.32	<.001	46 (28.6)		106 (65.8)	45 (28)	10 (6.2)
Potassium (mg)	0.39	<.001	42 (26.1)		106 (65.8)	45 (28)	10 (6.2)
Sodium (mg)	0.12	.12	46 (28.6)		116 (72)	33 (20.5)	12 (7.5)

^a^TEI: total energy intake.

### Categorization of Daily Nutrient Intakes

Cross-classification of quartiles is also presented in Table 4. The proportion of individuals remaining in the same quartile ranged from 22.9% (37/161) for total fat (% TEI) to 39.1% (63/161) for carbohydrates (g), with median of 32.3% (IQR 28.6%-34.5%). Of the 23 nutrients, 3 (13%) nutrients had extreme disagreement for ≤5% of participants (protein in both g and % TEI and sugar as % TEI), 17 (74%) had extreme disagreement for between >5% and ≤10% of participants, and 3 (13%) had extreme disagreement for >10% of participants (monounsaturated and polyunsaturated fat as % TEI and vitamin D in µg). When participants were classified according to nutrient-based guidelines (eg, low, adequate, or high) for the 14 nutrients, the proportion of participants who were classified into the same category by both methods ranged from 52.8% (85/161) for total fat (% TEI) to 84.5% (136/161) for protein (g/kg body weight; Table 5). The median proportion of participants who were classified correctly among the 14 nutrients was 70.5% (IQR 61.8%-78.9%).

**Table 5:** Percentage of participants classified to the same category when their mean daily nutrient intakes estimated from both the 24-hour recall and the novel, meal-based recall were categorized according to nutrient-based guidelines.

Nutrient	Possible categories for the classification of individual nutrient intakes	Participants classified to the same category (N=161), n (%)
Protein (g/kg of BW^a^)	Low, adequate, and high	136 (84.5)
Carbohydrate (% TEI^b^)	Low, adequate, and high	101 (62.7)
Total fat (% TEI)	Low, adequate, and high	85 (52.8)
Monounsaturated fat (% TEI)	Low, adequate, and high	128 (79.5)
Polyunsaturated fat (% TEI)	Low, adequate, and high	96 (59.6)
Saturated fat (% TEI)	Adequate and high	114 (70.8)
Salt (g)	Adequate and high	99 (61.5)
Dietary fiber (g)	Low and adequate	122 (75.8)
Calcium (mg)	Low, adequate, and high	92 (57.1)
Iron (mg)	Low, adequate, and high	135 (83.9)
Folate (µg)	Low, adequate, and high	133 (82.6)
Thiamin (mg)	Low and adequate	124 (77)
Riboflavin (mg)	Low and adequate	113 (70.2)
Vitamin C (mg)	Low, adequate, and high	106 (65.8)

^a^BW: body weight.

^b^TEI: total energy intake.

### Participant Evaluation Questionnaire

Most participants either somewhat agreed or agreed regarding generic recall that the instructions provided were clear and easy to understand (147/161, 91.3%); that the portion sizes in the tool were largely representative of the portion sizes they had consumed (137/161, 85.1%); and that, overall, the tool was easy to use (130/161, 80.7%). However, most (89/161, 55.3%) reported that the meal images were not representative of what they had actually consumed. This was anticipated, and for each meal in generic recall, participants were given the option to select that none of the meal images presented to them was similar to what they had consumed. This option was chosen for 36.04% (683/1895) of the total meals consumed, distributed across the meal types as follows: 17.4% (119/683) were breakfasts, 24.7% (169/683) were light meals, 20.4% (139/683) were main meals, 29.3% (200/683) were snacks, and 8.2% (56/683) were beverages. When these choices were reviewed by the researchers, by comparing participants’ text descriptions of their meals with the possible options from generic meal images, it was determined that 86.8% (593/683) of the meals were correctly recorded by participants as not having a matching generic meal, whereas 13.2% (90/683) of the meals could have been matched to one of the generic meal images. Although most of the participants (106/161, 65.8%) reported that the ease of use of the meal-based tool was worse or somewhat worse than the 24HR, 39.1% (63/161) of the participants reported that they would consider using a similar tool to generic recall again.

## Discussion

### Principal Findings

This study reports about a novel, meal-based recall that allows individuals to report their intakes of whole meals rather than reporting individual food or food group intakes for each meal, as is necessary in commonly used traditional dietary intake assessment methods. Estimated nutrient intakes obtained from the generic meal–based method were comparable with those obtained from the 24HR for some but not all nutrients. Comparisons between the methods were more similar at the group level than the individual level. Participants found the meal-based method understandable and easy to use. Previous studies have identified generic meals that exist in national dietary survey data [15,31-33], but this study is the first to use images of those generic meals as a novel method of dietary intake assessment.

In previous studies of generic meals, comparisons were made between the estimated nutrient intakes obtained from the original data and from the generic data. Agreement between the original and generic data was stronger in those studies than the agreement between the 2 methods of dietary assessment presented in this study; however, this was expected because those studies compared intakes arising from generic meals with intakes arising from the original data from which those generic meals were derived [15,31-33]. In this study, comparisons were made on data collected using 2 different methods; therefore, the generic meal images presented to participants were not influenced by those participants’ food intakes. Agreement between the methods in this study varied depending on the nutrient in question. In general, percentage differences and effect sizes for differences between the 2 methods were small for most nutrients (19/23, 83%). Certain nutrients showed poorer agreement than others including total fat, polyunsaturated fats, monounsaturated fats, and vitamin D. A few features of the generic method may have given rise to differences in these fat-soluble nutrients. In the food-based 24HR, participants could specify the types of fats that they added to foods; however, in the generic method, this was not the case, as participants had to choose between predefined generic meals. Some of these nutrients are found in relatively high concentrations in relatively few foods, which may also give rise to differences between a food-based method and a generic method. These trends have also been observed previously when comparing FFQs with 24HRs and food records, as noted by Cui et al [34] in a meta-analysis of 130 such studies. In the case of polyunsaturated fats, the difference between the median intakes was considerably larger than other nutrients. This was identified as having arisen from differences in food composition between the generic data and the 24HR data. The generic meals used in the generic recall and their nutritional composition were derived from data from NANS in Ireland [15,16]. The composition data in that survey, in turn, were obtained from a variety of sources including food packaging, industry information, published papers, and various food composition tables including those from the United Kingdom, Finland, and Australia that were published between 2002 and 2010 [35]. The composition data used in the 24HR were obtained from the 2021 publication of McCance and Widdowson’s CoFID [21]. Upon further examination of polyunsaturated fat content of individual foods from both NANS and CoFID, several foods were identified in NANS that had considerably higher values for polyunsaturated fat than those corresponding foods in CoFID in a manner which was not evident for other nutrients.

Although other studies have not examined the potential for dietary assessment based on individuals’ reporting of whole meal intakes, comparisons can be drawn with other related methods. Murakami et al [36] developed a meal-focused method called the food combination questionnaire (FCQ). In this method, participants reported, for each meal type (breakfast, lunch, dinner, and snacks), what staple foods they had consumed, what accompanying foods they had consumed with those staple foods, and how frequently per week they had consumed them during the preceding month. Agreement regarding estimates of nutrient intake between the FCQ and a 4-day weighed food record was similar to the agreement reported in this study for the comparison between the generic recall and 24HR. The median value of the statistically significant correlation coefficients was 0.35 compared with 0.31 in this study. In that study, however, the FCQ estimated lower intakes for most nutrients compared with food record, whereas in this study, the generic recall estimated higher intakes for most nutrients (16/23, 70%) compared with the 24HR.

Another approach to dietary intake assessment reported by Katz et al [37] involves participants reporting their intakes at the dietary pattern level instead of the meal, food group, or food levels. In this format, participants are presented with 2 images at a time. Each image contains multiple different foods and meals representing different dietary patterns based on the Healthy Eating Index, and the participant must choose which image is more representative of their dietary intakes. This process of choosing between 2 different diet pattern images is repeated until a “best fit” is identified for the individual [37]. Comparison of estimated nutrient intakes obtained from this method with those obtained from 3 separate 24HRs resulted in median correlation of 0.30 for correlations that were statistically significant [38]. Similar to this study, the abovementioned diet pattern approach also tended to estimate higher nutrient intakes than the 24HR. Similar trends were also observed in the Bland-Altman analysis with low bias or systematic error for macronutrients (ie, mean differences were close to 0) but slightly greater bias for micronutrients. Wide LOAs were also observed in both studies, indicating considerable random error. Random error can be reduced through repeated measures [39]; therefore, conducting >2 generic recalls performed in this study may mitigate against the random error observed here. Another trend that is evident from the Bland-Altman plots for macronutrients expressed as % TEI is that generic recall gives higher estimates of intake than 24HR when intakes are low and gives lower estimates of intake than 24HR when intakes are high. This arises from the narrowing of the distribution of intakes (ie, reduced variance) in the generic method, where participants can only choose from a small number of generic meals compared with the practically limitless different combinations of foods that participants can choose from in 24HR. The use of portion sizes mitigates against this trend for values expressed in absolute intakes, for example, grams of macronutrient intake. However, expressing those values relative to EI effectively nullifies the increased variance introduced by portion size, giving rise to the trends observed in the Bland-Altman plots for values given as % TEI but not for those given as absolute intakes.

A version of FCQ described previously has been used to provided dietary advice in a pilot study by Murakami et al [40]. Similarly, the dietary pattern–based method of dietary assessment described previously has also been incorporated into a commercially available tool that provides nutrition recommendations to users [41]; however, the recommendation aspect of this tool has not been described in the scientific literature. It has also been proposed that a meal-based method of dietary intake assessment could provide a low-burden alternative to gathering dietary data for personalized nutrition advice [3]. This study presents the first attempt at implementing such a proposal. Its performance in estimating nutrient intakes is similar to those of other approaches that focus on meals or dietary patterns. However, these methods tend to have poorer agreement compared with that between FFQs and 24HRs, where median correlation of 0.42 has been reported for the various nutrients assessed [34], or compared with the agreement between 24HRs and diet records, where median correlations in the range of 0.45 to 0.50 have been reported [42,43]. Further studies are required to appraise the performance of these methods, including generic recall, against objective biomarkers of dietary intake.

Many of the comparisons between the 2 methods in this study are based on their ability to provide point estimates of nutrient intakes, with agreement for point estimates of nutrient intake typically stronger at the group level than at the individual level. This trend has also been observed in comparisons of other methods of dietary intake assessment, for example, between 24HR and diet record [42], between FFQ and diet record [44], and between FFQ and 24HR [45]. In this study, this was expected, given that there are fewer generic meals than foods for participants to choose from, that is, the variance was intentionally reduced but in a manner that was systematic and consistent across meals [15]. This trend has been observed not just in comparison studies of different methods of dietary assessment but also in studies that have used generic meals as a method for secondary analysis of dietary data that have already been collected [15,31-33]. Approaches to personalized nutrition facilitated by technology, however, do not rely solely on point estimates of nutrient intake. Instead, individuals are categorized into ranges of nutrient intakes (eg, low, adequate, or high), allowing room for error in the point estimates [40,46]. In the Food4Me study of personalized nutrition, for example, participants were categorized in this manner, and dietary advice reports were tailored depending on the participants’ categories for various nutrients [40,46]. This study has shown that individuals can be classified according to nutrient-based dietary guidelines using generic recall. Similarly, this approach can be used to rank individuals based on their nutrient intakes, with values from the cross-classification of quartiles being comparable with those observed in studies comparing FFQs with 24HRs, where median exact + adjacent agreement has been reported between 66% and 86.1% [47-49].

Notably, with regard to the use of generic recall in dietary assessment in personalized nutrition, most participants (130/161, 80.7%) reported in the evaluation questionnaire that the recall was easy to use. Ease of use has previously been reported as an important factor in the choice of nutrition or diet apps among a cohort of 2382 adults in Europe [5]. In contrast, two-thirds of the participants reported that the ease of use of the 24HR was better than that of the meal-based approach. This is understandable given that the web-based 24HR used in this study was developed as a stand-alone platform in collaboration with software developers [20], whereas generic recall was a concept or pilot tool implemented by the authors using a commercially available questionnaire platform and not specifically designed for user experience. Future user evaluations and collaboration with software development professionals could further enhance the user experience of generic recall, including incorporation of meal image recognition.

So far, many studies have been conducted on image-based food recognition using computer vision as a means of reducing the burden of data input for food-based methods of dietary intake assessment [13]. These image recognition tools, however, are food based, insofar as when an individual takes a photo of their meal, the software segments the image and provides a suggested match for each of the individual foods that make up the meal [13]. The user must then confirm that each of the suggested foods are correct. For any missing or incorrect foods, the user must text search for the correct food and add it to their record [12]. It is possible that a meal-based approach could be taken to image recognition in dietary assessment, removing the need to identify individual foods. Instead, the software would classify a whole meal image as one of the generic meals used in this study. Further studies are required, however, to determine the feasibility of such an approach for meal-based image recognition in nutrition.

### Limitations

This study has a number of limitations. The generic meal images used are based on dietary intake data obtained from NANS (2008-2010) [16]. Although, at the time of writing, these were the most recently published intake data in Ireland, the generic meals do not account for any changes in food composition or meal intakes that may have occurred since that time. This may account for the findings in the evaluation questionnaire of this study, with 55.3% (89/161) of the participants reporting that the generic meals were not representative of their intakes, and shows the need to ensure that generic meals are revised using most recent data. This may be of particular importance if the tool was to be used with a younger cohort of participants than those who participated in this study. This study also used a convenience sample rather than one that is representative of the population in Ireland. This may result in the recruitment of participants who are more likely to have an interest in nutrition and health. The unbalanced nature of the demographics of the study participants precludes subgroup analysis in relation to demographic factors.

The 2 types of recall used in this study are influenced by measurement error and only provide an estimation of true intakes. This study aimed to compare these 2 methods, and therefore, the reported statistics should be interpreted as representing the relationship between generic recall and 24HR and not the relationship between generic recall and true intakes. A comparison study was deemed more appropriate as an initial indication of generic recall’s strengths and weaknesses before considering more labor-intensive and costly objective measures of comparison such as feeding studies or biomarkers of dietary intake.

### Strengths

The strengths of this study include its sample size of 161 participants. The randomization of participants regarding the recall method they would complete first, the reversal of the order of completion in their second set of recalls, and the 2-week washout period mitigates against any learning effect that participants may have experienced after completing the first recall. The comparison method used, Foodbook24, is a validated, web-based 24HR [20]. This study also captured dietary intakes on both weekend days and weekdays, accounting for the potential differences in eating habits that occur between the 2 categories [50-52].

### Conclusions

A generic meal–based method of dietary intake assessment provides estimates of nutrient intake comparable with those provided by a web-based 24HR. The agreement ranges among nutrients from weak to moderate, with better agreement at the group level than the individual level. Further studies are required to improve this method of dietary assessment considering the number of recalls required, and more recent dietary intake data should be used to define the generic meals. Future studies will determine the feasibility of taking a meal-based approach to image recognition in dietary intake assessment.

## Appendix

Multimedia Appendix 1 Analysis of the smaller data set, with misreporters excluded.

## Abbreviations

24HR: 24-hour recall
BMR: basal metabolic rate
CoFID: Composition of Foods Integrated Dataset
EI: energy intake
FCQ: food combination questionnaire
FFQ: food frequency questionnaire
LOA: limit of agreement
NANS: National Adult Nutrition Survey
NRF: Nutrient Rich Foods
TEI: total energy intake

## Data Availability

The data sets generated and analyzed during this study are available from the corresponding author upon reasonable request.
