# Supplementary material for: Dietary Intake Assessment Using a Novel, Generic Meal–Based Recall and a 24-Hour Recall: Comparison Study
Source: J Med Internet Res. 2024 Feb 14;26:e48817. doi: 10.2196/48817 (PMC10902769; doi:10.2196/48817)
Supplement: Multimedia Appendix 1 [file jmir_v26i1e48817_app1.docx]

Multimedia Appendix 1

Table S1. Daily nutrient intakes estimated using a web-based 24-hour recall and the novel generic meal-based recall (n = 108), with misreporters of energy intake removed.^a, b, c^

|  | Recall Method | | % Difference | *P* value | Effect Size (*r*) | Effect Size  (Magnitude) |
| --- | --- | --- | --- | --- | --- | --- |
|  | 24HR  Median (IQR) | Generic  Median (IQR) |  |  |  |  |
| Energy (kcal) | 1807.7 (1513.1–2148.7) | 1744.9 (1508.8–1963.5) | -3.5 | 0.080 | 0.168 | small |
| Protein (g) | 75.4 (60.4–93.2) | 72.9 (65.2–83) | -3.3 | 0.027 | 0.213 | small |
| Protein (% TEI) | 16.3 (13.8–19.6) | 16.4 (15.5–17.9) | 0.3 | 0.707 | 0.036 | small |
| Carbohydrate (g) | 209.4 (181–264.2) | 209.9 (180.8–244.1) | 0.2 | 0.470 | 0.070 | small |
| Carbohydrate (% TEI) | 45.4 (39.4–50.4) | 45.4 (43.7–47.4) | 0.0 | 0.165 | 0.134 | small |
| Sugars (g) | 84.6 (62.6–110.5) | 89 (70–104.5) | 5.2 | 0.327 | 0.094 | small |
| Sugars (% TEI) | 17 (13.5–21.9) | 19.1 (17.5–21.7) | 12.7 | 0.015 | 0.235 | small |
| Dietary Fibre (g) | 18.3 (14.2–23.5) | 16.8 (15–19.9) | -8.1 | 0.002 | 0.293 | small |
| Total Fat (g) | 70.8 (57.9–83.6) | 63.5 (54.5–75.7) | -10.3 | 0.005 | 0.271 | small |
| Total Fat (% TEI) | 34.5 (30–39.6) | 33.6 (31.4–35.4) | -2.7 | 0.036 | 0.202 | small |
| Saturated Fat (g) | 24.9 (19–33.7) | 25.3 (21.9–30.5) | 1.5 | 0.923 | 0.009 | small |
| Saturated Fat (% TEI) | 12.4 (9.5–15.3) | 13.4 (12.9–14.1) | 7.9 | 0.134 | 0.145 | small |
| Monounsaturated Fat (g) | 25.3 (20–31.1) | 23.1 (19.7–27) | -8.9 | 0.005 | 0.268 | small |
| Monounsaturated Fat (% TEI) | 12.4 (10.6–14.6) | 12.1 (11.2–12.8) | -2.5 | 0.099 | 0.159 | small |
| Polyunsaturated Fat (g) | 8.6 (6.5–10.2) | 11.4 (9.4–12.9) | 32.9 | 0.000 | 0.497 | moderate |
| Polyunsaturated Fat (% TEI) | 4.1 (3.4–4.9) | 5.9 (5.3–6.2) | 41.7 | 0.000 | 0.766 | large |
| Vitamin D (µg) | 2.2 (0.8–4.2) | 2.4 (2–2.6) | 6.1 | 0.180 | 0.129 | small |
| Vitamin E (mg) | 8.3 (6.5–11) | 8.2 (6.7–9.3) | -1.6 | 0.036 | 0.201 | small |
| Folate (µg) | 234.2 (187.7–289) | 200.4 (174.4–226.6) | -14.4 | 0.000 | 0.477 | moderate |
| Vitamin C (mg) | 89.1 (46.4–147.3) | 67.8 (56.1–80.9) | -23.9 | 0.000 | 0.413 | moderate |
| Calcium (mg) | 766.5 (597.7–965.3) | 822.1 (714.6–948.5) | 7.3 | 0.233 | 0.115 | small |
| Magnesium (mg) | 298.9 (253.4–358.1) | 258.3 (218.8–285.9) | -13.6 | 0.000 | 0.571 | large |
| Phosphorous (mg) | 1272.5 (1019.3–1493.3) | 1218.3 (1079.5–1365.6) | -4.3 | 0.037 | 0.201 | small |
| Iron (mg) | 10.9 (9.1–13.6) | 10.2 (8.4–12.1) | -6.6 | 0.001 | 0.327 | moderate |
| Potassium (mg) | 3109.9 (2464.4–3726.7) | 2763.5 (2343.9–3089.4) | -11.1 | 0.000 | 0.435 | moderate |
| Sodium (mg) | 1917.9 (1502.3–2381.7) | 2162.1 (1909–2444.7) | 12.7 | 0.029 | 0.210 | small |

^a^ *P*-values were derived using Wilcoxon’s Signed Rank Test, with *P* < 0.05 indicating statistical significance.

^b^ An effect size of ≥ 0.1 and < 0.3 was considered small, ≥ 0.3 and <0.5 was considered moderate, and ≥ 0.5 was considered large.

^c^ Energy misreporters were defined as those with an energy intake to basal metabolic rate ratio of less than 0.96 or greater than 2.49.

Table S2. Bland-Altman analysis of nutrient intake estimates arising from a web-based 24-hour recall and the generic recall (n = 108), with misreporters of energy intake removed.^a, b^

|  | Mean Difference | Lower LOA | Upper LOA | Percentage within LOA |
| --- | --- | --- | --- | --- |
|  |  |  |  |  |
| Energy (kcal) | 123.2 | -892.8 | 1139.3 | 96.3 |
| Protein (g) | 6.6 | -45.8 | 59.1 | 95.4 |
| Protein (% TEI) | 0.3 | -7.6 | 8.1 | 95.4 |
| Carbohydrate (g) | 9.0 | -120.1 | 138.1 | 93.5 |
| Carbohydrate (% TEI) | -1.2 | -15.4 | 13.1 | 95.4 |
| Sugars (g) | -0.6 | -81.8 | 80.5 | 93.5 |
| Sugars (% TEI) | -1.2 | -13.3 | 10.9 | 95.4 |
| Dietary Fibre (g) | 2.4 | -11.5 | 16.3 | 95.4 |
| Total Fat (g) | 8.5 | -44.5 | 61.5 | 93.5 |
| Total Fat (% TEI) | 1.7 | -12.5 | 15.9 | 97.2 |
| Saturated Fat (g) | 0.8 | -24.5 | 26.0 | 94.4 |
| Saturated Fat (% TEI) | -0.6 | -8.9 | 7.8 | 97.2 |
| Monounsaturated fat (g) | 3.2 | -17.3 | 23.7 | 95.4 |
| Monounsaturated Fat (% TEI) | 0.6 | -5.5 | 6.7 | 95.4 |
| Polyunsaturated fat (g) | -2.3 | -10.7 | 6.1 | 93.5 |
| Polyunsaturated Fat (% TEI) | -1.5 | -4.3 | 1.2 | 93.5 |
| Vitamin D (µg) | 0.8 | -5.4 | 7.0 | 96.3 |
| Vitamin E (mg) | 1.0 | -7.1 | 9.0 | 95.4 |
| Folate (µg) | 56.9 | -159.7 | 273.4 | 93.5 |
| Vitamin C (mg) | 38.0 | -120.5 | 196.5 | 95.4 |
| Calcium (mg) | 0.9 | -766.4 | 768.2 | 95.4 |
| Magnesium (mg) | 58.5 | -115.0 | 232.0 | 93.5 |
| Phosphorous (mg) | 107.8 | -761.3 | 976.9 | 96.3 |
| Iron (mg) | 1.4 | -6.0 | 8.8 | 93.5 |
| Potassium (mg) | 486.2 | -1475.8 | 2448.2 | 94.4 |
| Sodium (mg) | -124.0 | -2048.0 | 1800.0 | 92.6 |

^a^ Differences are given as values from the 24HR minus values from the generic recall.

^b^ Energy misreporters were defined as those with an energy intake to basal metabolic rate ratio of less than 0.96 or greater than 2.49.

%TEI: % Total Energy Intake.

Table S3. Comparison of daily nutrient intakes estimated using a web-based 24-hour recall and the novel meal-based method, based on correlation and cross-classification of quartiles (n = 108), with misreporters of energy intake removed^a^.

|  | Correlation | |  | Cross-Classification of Quartiles | | | |
| --- | --- | --- | --- | --- | --- | --- | --- |
|  | Spearman Coefficient | *P* |  | Exact Agreement (%) | Exact Agreement + Adjacent (%) | Disagreement (%) | Extreme Disagreement (%) |
| Energy (kcal) | 0.35 | 0.000 |  | 38.0 | 74.1 | 19.4 | 6.5 |
| Protein (g) | 0.21 | 0.027 |  | 32.4 | 63.9 | 28.7 | 7.4 |
| Protein (% TEI) | 0.42 | 0.000 |  | 30.6 | 77.8 | 19.4 | 2.8 |
| Carbohydrate (g) | 0.45 | 0.000 |  | 34.3 | 78.7 | 15.7 | 5.6 |
| Carbohydrate (% TEI) | 0.47 | 0.000 |  | 38.0 | 82.4 | 12.0 | 5.6 |
| Sugars (g) | 0.40 | 0.000 |  | 38.9 | 77.8 | 16.7 | 5.6 |
| Sugars (% TEI) | 0.43 | 0.000 |  | 34.3 | 77.8 | 19.4 | 2.8 |
| Dietary Fibre (g) | 0.47 | 0.000 |  | 35.2 | 79.6 | 13.0 | 7.4 |
| Total Fat (g) | 0.20 | 0.035 |  | 33.3 | 72.2 | 18.5 | 9.3 |
| Total Fat (% TEI) | 0.17 | 0.087 |  | 26.9 | 66.7 | 25.0 | 8.3 |
| Saturated Fat (g) | 0.19 | 0.050 |  | 31.5 | 70.4 | 20.4 | 9.3 |
| Saturated Fat (% TEI) | 0.14 | 0.139 |  | 25.0 | 65.7 | 26.9 | 7.4 |
| Monounsaturated fat (g) | 0.18 | 0.066 |  | 30.6 | 70.4 | 23.1 | 6.5 |
| Monounsaturated Fat (% TEI) | 0.09 | 0.356 |  | 27.8 | 64.8 | 24.1 | 11.1 |
| Polyunsaturated fat (g) | 0.13 | 0.175 |  | 26.9 | 64.8 | 25.0 | 10.2 |
| Polyunsaturated Fat (% TEI) | 0.22 | 0.023 |  | 28.7 | 73.1 | 17.6 | 9.3 |
| Vitamin D (µg) | 0.11 | 0.256 |  | 29.6 | 68.5 | 14.8 | 16.7 |
| Vitamin E (mg) | 0.27 | 0.006 |  | 29.6 | 72.2 | 20.4 | 7.4 |
| Folate (µg) | 0.30 | 0.002 |  | 23.1 | 68.5 | 23.1 | 8.3 |
| Vitamin C (mg) | 0.26 | 0.006 |  | 27.8 | 64.8 | 25.9 | 9.3 |
| Calcium (mg) | 0.19 | 0.044 |  | 31.5 | 66.7 | 22.2 | 11.1 |
| Magnesium (mg) | 0.31 | 0.001 |  | 34.3 | 72.2 | 19.4 | 8.3 |
| Phosphorous (mg) | 0.24 | 0.014 |  | 25.0 | 71.3 | 23.1 | 5.6 |
| Iron (mg) | 0.27 | 0.005 |  | 24.1 | 66.7 | 25.9 | 7.4 |
| Potassium (mg) | 0.34 | 0.000 |  | 25.9 | 67.6 | 22.2 | 10.2 |
| Sodium (mg) | 0.12 | 0.202 |  | 24.1 | 60.2 | 27.8 | 12.0 |

^a^ Energy misreporters were defined as those with an energy intake to basal metabolic rate ratio of less than 0.96 or greater than 2.49.

Table S4. Percentage of participants classified to the same category when their mean daily nutrient intakes estimated from both the 24-hour recall and the novel meal-based recall were categorised according to nutrient-based guidelines (n = 108), with misreporters of energy intake removed.^a^

| Nutrient | Possible categories for classification of individual nutrient intakes | % classified to the same category |
| --- | --- | --- |
| Protein (g/kg BW) | Low, adequate, and high | 89.8 |
| Carbohydrate (% TEI) | Low, adequate, and high | 68.5 |
| Total Fat (% TEI) | Low, adequate, and high | 54.6 |
| Monounsaturated Fat (% TEI) | Low, adequate, and high | 81.5 |
| Polyunsaturated Fat (% TEI) | Low, adequate, and high | 66.7 |
| Saturated Fat (% TEI) | Adequate and high | 71.3 |
| Salt (g) | Adequate and high | 76.9 |
| Dietary Fibre (g) | Low and adequate | 73.1 |
| Calcium (mg) | Low, adequate, and high | 55.6 |
| Iron (mg) | Low, adequate, and high | 88.0 |
| Folate (µg) | Low, adequate, and high | 81.5 |
| Thiamin (mg) | Low and adequate | 80.6 |
| Riboflavin (mg) | Low and adequate | 78.7 |
| Vitamin C (mg) | Low, adequate, and high | 64.8 |

^a^ Energy misreporters were defined as those with an energy intake to basal metabolic rate ratio of less than 0.96 or greater than 2.49.
